# 3D bioprinting of integral ADSCs-NO hydrogel scaffolds to promote severe burn wound healing

**DOI:** 10.1093/rb/rbab014

**Published:** 2021-04-25

**Authors:** Yu Wu, Tangzhao Liang, Ying Hu, Shihai Jiang, Yuansen Luo, Chang Liu, Guo Wang, Jing Zhang, Tao Xu, Lei Zhu

**Affiliations:** 1 Department of Plastic and Aesthetic Surgery, The Third Affiliated Hospital of Sun Yat-sen University, No. 600 Tianhe Road, Tianhe District, Guangzhou 510630, China; 2 East China Institute of Digital Medical Engineering, Shangrao 334000, China; 3 Department of Joint and Trauma Surgery, The Third Affiliated Hospital of Sun Yat-sen University, Guangzhou 510630, China; 4 Institute of Laboratory Medicine, Clinical Chemistry and Molecular Diagnostics, University Hospital Leipzig, Leipzig 04103, Germany; 5 Department of The Second Plastic Surgery, The First People’s Hospital of Foshan, Foshan 528000, China; 6 Department of Mechanical Engineering, Tsinghua University, No. 30 Shuangqing Road, Haidian District, Beijing 100084, China; 7 Tsinghua-Berkeley Shenzhen Institute, Tsinghua University, Shenzhen 518055, China

**Keywords:** 3D bioprinting, ADSCs, nitric oxide, angiogenesis, severe burn, wound healing

## Abstract

Severe burns are challenging to heal and result in significant death throughout the world. Adipose-derived mesenchymal stem cells (ADSCs) have emerged as a promising treatment for full-thickness burn healing but are impeded by their low viability and efficiency after grafting *in vivo*. Nitric oxide (NO) is beneficial in promoting stem cell bioactivity, but whether it can function effectively *in vivo* is still largely unknown. In this study, we bioprinted an efficient biological scaffold loaded with ADSCs and NO (3D-ADSCs/NO) to evaluate its biological efficacy in promoting severe burn wound healing. The integral 3D-ADSCs/NO hydrogel scaffolds were constructed via 3D bioprinting. Our results shown that 3D-ADSCs/NO can enhance the migration and angiogenesis of Human Umbilical Vein Endothelial Cells (HUVECs). Burn wound healing experiments in mice revealed that 3D-ADSCs/NO accelerated the wound healing by promoting faster epithelialization and collagen deposition. Notably, immunohistochemistry of CD31 suggested an increase in neovascularization, supported by the upregulation of vascular endothelial growth factor (VEGF) mRNA in ADSCs in the 3D biosystem. These findings indicated that 3D-ADSC/NO hydrogel scaffold can promote severe burn wound healing through increased neovascularization via the VEGF signalling pathway. This scaffold may be considered a promising strategy for healing severe burns.

## Introduction

Burns are the fourth most common type of trauma worldwide and cause ∼265 000 deaths every year [1, 2]. Severe burn injuries may result in both physical and psychological harm in patients [3]. Early burn wound excision and autologous skin grafting are the most common treatments for severe or large-area burn repair [4, 5]. However, limitations on available healthy skin remain a major challenge for skin regeneration [2]. Therefore, an efficient treatment for severe burn wounds is urgently needed.

In recent years, adipose-derived mesenchymal stem cell (ADSCs) transplantation has been reported as a potential treatment for wound healing [6]. ADSCs can be easily obtained from liposuction of human adipose tissue and effectively exert biological functions *in vitro* [7, 8]. ADSCs have been proven to reduce the scar tissue area and increase collagen Type III deposition in burn wound healing [9]. In addition, ADSCs have been permitted to be used in clinical and achieved very good surgical outcomes and patient satisfaction in burn wound [10]. Our previous study showed that ADSCs are likely attributed to paracrine secretion of trophic factors rather than direct differentiation [11]. However, low cellular retention, survival rate and engraftment after *in vivo* transplantation have limited translational medicine [12].

NO has become an attractive candidate for its ability to regulate inflammation and angiogenesis in wound healing therapy [13, 14]. In many chronic ulcers, and especially in patients with diabetes, the continuous supplement of NO improves healing process [15]. S-Nitroso-N-acetyl-D, L-penicillamine (SNAP), as a NO donor, plays a protective role against ischaemia/reperfusion injury and enhances the proangiogenic potential of mesenchymal stem cells [16]. However, its possible therapeutic application is limited due to its extremely short half-life and low bioavailability [17]. Suitable carriers for NO delivery seem to be critical for exerting maximal efficacy [18]. Previous studies have shown that a simple NO scaffold has become a therapeutic strategy to promote angiogenic activity in endothelial cells [14, 19]. Therefore, it is possible that a gelatine-sodium (GS) alginate hydrogel loaded with SNAP emerge as suitable carrier.

For the last few years, 3D bioprinting has occupied an important position as an advanced technology that can address many problems faced in conventional tissue engineering [20]. Extrusion-based bioprinting, one of the most interesting techniques that has been explored, extrudes continuous fibres of biomaterials to form a 3D scaffold structure [21]. Extrusion-based bioprinting can precisely produce tissue scaffolds in a controlled manner using layer-by-layer deposited hydrogels and suspended cells, unlike other bioprinting technologies [22]. Moreover, culturing cells in a 3D construct is more suitable for simulating the microenvironment and subsequently influencing behaviour and gene expression [23]. Recent studies have intended to improve stem cell retention at wound sites by using spheroids, hydrogel systems, and biological scaffolds [24–26]. At present, few studies have focussed on the combination of stem cells and nitric oxide (NO) in 3D bioscaffolds.

**Figure 1.**
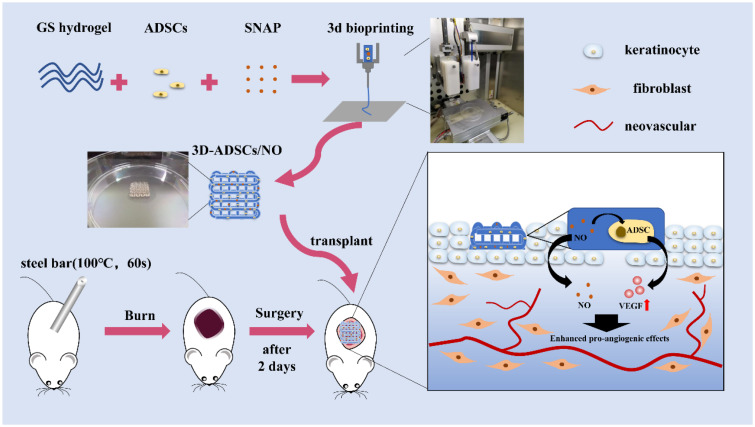
Schematic illustration of 3D-ADSCs/NO for promoting burn wound healing.

Herein, a highly efficient GS hydrogel loaded with ADSCs and NO was constructed by 3D bioprinting to evaluate its efficiency in promoting severe burn wound healing. The main objective was to evaluate the effects of 3D-ADSCs/NO on promoting angiogenesis and severe burn wound healing both *in vitro* and *in vivo* (Fig. 1).

## Materials and methods

### Isolation and culture of adipose-derived stem cells

Isolation of ADSCs was performed according to our previous report [27]. Briefly, inguinal subcutaneous fat was collected from Balb/c mice (male, 8 weeks old) and minced into pieces <1 mm in diameter before digestion with 0.1% Type I collagenase (Gibco, 17100017) at 37°C for 45 min. Afterward, the digestate was neutralized by Dulbecco's modification of Eagle's medium (DMEM) containing 10% FBS and passed through a 70-μm filter to remove undigested tissue. The cells were collected and seeded into T-25 flasks at a final concentration of 5 × 10^6^ cells/ml. The cells were cultured in a 37°C incubator supplied with 5% CO_2_ and 95% humidity. The cell culture medium was changed every 2 days. The third- to fifth-passage cells were used for subsequent experiments. We had identified the ADSCs through flow cytometry analysis and differentiation abilities analysis in our previous study [27].

### 3D construct bioprinting

For the formation of GS alginate hydrogels [28], gelatine (Aladdin, G108395) and sodium alginate (Aladdin, S100128) were dissolved in normal saline (NS) at concentrations of 20% and 2% (w/v). The hydrogels underwent high-pressure steam sterilization. ADSCs were collected and resuspended in culture medium. Then, we gently mixed the cell supernatant with GS hydrogels to reach a final concentration of 2 × 10^6^ cells/ml, 10% gelatine, and 1% sodium alginate. Hydrogels were additionally loaded with 5‰ SNAP (Aladdin, S131283). The mixture was then loaded in a 1 ml syringe and set on an extrusion bioprinter (Livprint norm, Medprin, Guangzhou, China). The square grid scaffolds were designed with 15 × 15 mm cross sectional area and 2-mm thickness. The printing chamber was set to 4°C, pressure was set to 0.2 mPa, 0.26 mm diameter nozzle was selected, and the scanning speed was controlled at 8 mm/s. The printing pathway was grid-like and each layer had eight microwires. Bioprinted 3D constructs were immediately immersed in 3% sterile calcium chloride solution for 3 min for crosslinking. The constructs were cultured in a 6-well plate under the same conditions as the ADSCs.

### Scanning electron microscopy analysis

The 3D-ADSCs/NO were immersed in 2.5% glutaraldehyde for 1 h for fixation. Samples were dehydrated using graded ethanol (50%, 70%, 80%, 90%, 95% and 100%) and lyophilized in a freeze drier for 12 h to eliminate residual water. All samples were sputter-coated with platinum and imaged with an ULTRA 55 scanning electron microscope (SEM; ZEISS, Germany).

### *In vitro* NO release

NO release was measured by using the Griess assay as described in previous research [14]. Specifically, GS hydrogels loaded with SNAP (3D-NO) were immersed absolutely in a sterile saline solution at room temperature. Fifty microlitres of supernatant was collected at every point of time (1, 2, 4, 8 h, 1, 2, 3, 4 and 5 days) and supplemented with the same volume of saline solution. The samples were transferred into a 96-well plate and treated with Griess reagent (Beyotime, S0021S) by adding 50 μl to each well. The absorbance was measured at a wavelength of 540 nm in a microplate reader. The recorded absorbances were then converted into NO concentrations using the standard calibration curve shown in Supplementary Fig. S1A.

### Live–dead assay

A live–dead cell staining kit (KeyGEN, KGAF001) was used to determine the effect of NO on ADSC viability and mortality according to the manufacturer’s protocol [28]. Images were taken with a fluorescence microscope (Mshot, China) at varying resolutions.

### Alamar blue

Cell proliferation of 3D-ADSCs and 3D-ADSCs/NO was detected using the Alamar Blue Kit (Thermo Fisher, DAL1025). Briefly, 0.1 mM Alamar blue working solution was prepared by 10× dilution of 1 mM sterile Alamar blue stock solution with DMEM. At each time point (1, 3 and 5 days), 2 ml of Alamar blue working solution was added to each well and incubated for 4 h at 37°C in the dark. After incubation, 100 μl of supernatant from each well was transferred to a 96-well plate, and the optical density (OD) value was read at 570 and 630 nm wavelengths. OD values from both groups were normalized to Day 1 for plotting and statistics.

### Transwell assay

HUVECs were cultured in DMEM supplemented with 10% FBS in a humidified atmosphere containing 5% CO_2_ at 37°C. A total of 1 × 10^4^ HUVECs were added to each Transwell (Corning, 3422) upper chamber, and 600 μl of fresh medium or culture supernatant containing 10% FBS was placed in the lower chamber [29]. HUVECs were treated with supernatant from 2D-ADSCs, 3D-ADSCs- or 3D-ADSCs/NO for 24 h, and the group without supernatant was the control group. The cells were incubated for 24 h for the migration assay. After 24 h, the cells that migrated through the basolateral membrane were fixed with 4% paraformaldehyde and stained with 0.1% crystal violet. Images of invaded HUVECs were observed and collected by an optical microscope (Mshot, China).

### Tubule formation assay

The tube formation assay was conducted using HUVECs according to the published literature [30]. A total of 1 × 10^4^ HUVECs were seeded onto Matrigel (BD, 356234) in 96-well plates and incubated in serum-free cell culture supernatant 2D-ADSCs, 3D-ADSCs and 3D/NO-ADSCs or DMEM (control) at 37°C. Tube formation was examined 8 h later. The number of meshes, master segments, master junctions, and total master segment length were measured by ImageJ (NIH, Bethesda, MD).

### Quantitative real-time polymerase chain reaction (qRT-PCR)

Total RNA of ADSCs in 3D constructs was extracted using an RNA Quick Purification kit (ESscience, RN001) and stored at −80°C. Then, the qRT-PCR assay was performed using the One Step TB Green^®^ PrimeScript™ RT-PCR Kit II (TAKARA, RR086A) according to the manufacturer’s instructions. The primers used in qRT-PCR were as follows:

vascular endothelial growth factor (VEGF), forward: 5′-CCACGACAGAAGGAGAGCAGAAG-3′ and

reverse: 5′- ACAGGACGGCTTGAAGATGTACTC-3′;

Glyceraldehyde-3-phosphate dehydrogenase (GAPDH), forward: 5′- AGGTCGGTGTGAACGGATTTG-3′ and

reverse: 5′- TGTAGACCATGTAGTTGAGGTCA -3′.

The qRT-PCR assay was performed using the LightCycler 480 Real-Time Fluorescence PCR system.

### *In vivo* burn wound healing

All animal experiments were performed in strict accordance with the Third Affiliated Hospital of Sun Yat-sen University medical ethics committee guidelines. Burn wound models were created on 32 Balb/c mice (male, 8 weeks old) according to an established model [31, 32]. The mice were anaesthetized by 50 mg/kg sodium pentobarbital through intraperitoneal injection. Dorsal hair was removed, and full-thickness burn wounds were made using steel bars (diameter: 14 mm, 100°C, 1 min). The necrotic skin was removed after 48 h, and a silicon ring was sutured surrounding the wound area (Fig. 4A). The mice were randomly divided into four groups according to different treatments after surgery: control group, 3D-ADSCs, 3D-NO and 3D-ADSCs/NO. Gauze was applied to the control group. The hydrogel constructs were applied once after the wound was generated. Then, the wounds were covered with a 3 M Tegaderm and gauze. Wound images were taken at scheduled time points (0, 7 and 14 days), and the wound area was analysed by ImageJ software. The wound area was calculated according to the following equation:

$$\mathrm{Wound}\;\mathrm{area}\left(\%\right)=\frac{\mathrm{residual}\;\mathrm{size}}{\mathrm{original}\;\mathrm{size}}\times 100\%$$

wound tissues were harvested at seven days (*n* = 2) and 14 days (*n* = 6). Each sample was immersed in a 4% formaldehyde solution for histological tests.

### Histochemistry and immunohistochemistry

For histochemistry and immunohistochemistry analysis, the wound tissues were fixed in 4% formaldehyde solution and dehydrated with ethanol. Then, tissues were embedded in paraffin and sectioned into slices perpendicular to the wound surface. The tissue sections were stained with haematoxylin and eosin (HE) and Masson’s trichrome (MT) following conventional protocols. HE staining was used to examine new tissue formation and infiltrated cells. MT staining was used to detect collagen deposition. An upright optical microscope captured the images.

To assess the angiogenesis of different groups, immunohistochemical staining of CD31 was performed. Briefly, sodium citrate was used to repair tissue antigens for 15 min. The tissue was then blocked with 3% Bull Serum Albumin (BSA) for 30 min and incubated with anti-CD31 (1:100, Abcam) at 4°C overnight. The Horseradish Peroxidase (HRP) -conjugated secondary antibody was added on the second day for 50 min. Then, the nucleus was stained with haematoxylin. Finally, the slices were imaged using an upright optical microscope and analysed by ImageJ.

### Statistical analysis

The data from NO release, the Alamar blue assay, Transwell assay, cell tubule formation assay *in vitro*, and burn wound healing *in vivo* are expressed as the mean ± SD. Student’s t-test was performed for statistical comparisons between the two groups. One-way analysis of variance was performed to statistically compare differences among multiple groups. A *P* value < 0.05 was considered to be statistically significant.

## Results and discussion

### Establishment of the 3D-ADSC/NO hydrogel constructs

**Figure 2.**
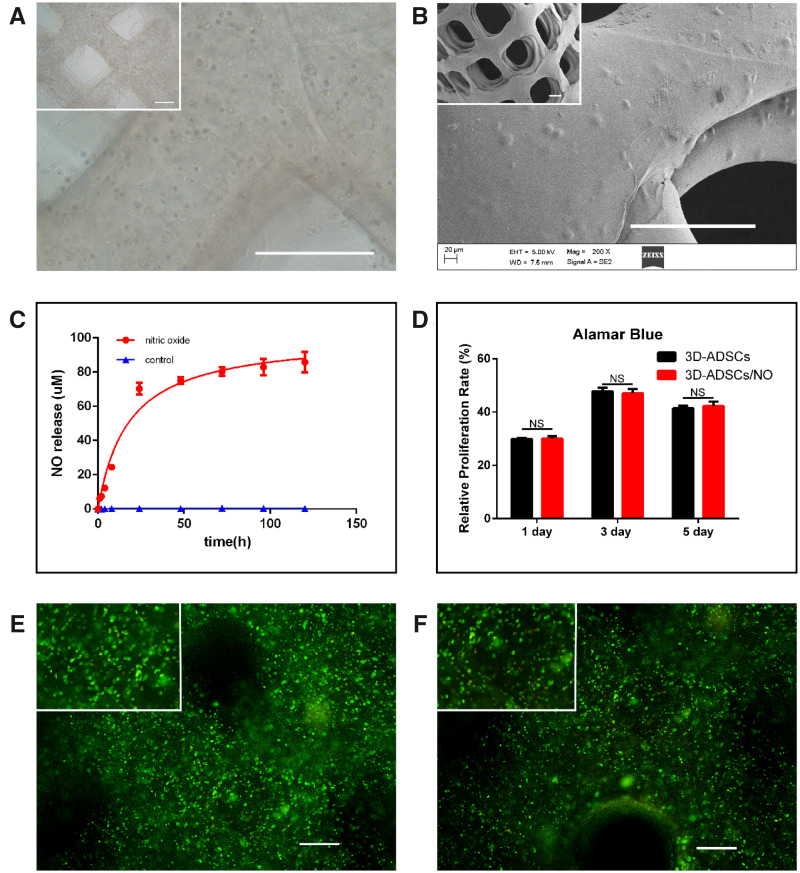
(**A**) Microscopy images of 3D-ADSC/NO constructs showed that ADSCs were uniformly distributed within the hydrogel (scale bar: 500 µm). (**B**) SEM images of 3D-ADSC/NO confirmed that the grid-like constructs and ADSCs grew both inside the hydrogel and on the surface of the hydrogel. (scale bar: 500 µm). (**C**) A cumulative NO release study of 3D-NO exhibited a burst release within 4 h and sustained release even after 120 h. (**D**) Alamar blue assay in 3D-ADSCs and 3 D-ADSCs/NO. The cell proliferation rate increased over time but slowly decreased after 5 days and no significant difference was found between 3D-ADSCs and 3D-ADSCs/NO (NS, no significant difference). Representation Of cell viability and mortality in 3D-ADSCs/NO (**E**) and 3D-ADSCs (**F**) by using a live-dead assay. There was no significant difference in cell viability between 3D-ADSCs/NO and 3D-ADSCs (scale bar: 100 µm).

Gelatine-based biomaterials have been widely used in tissue engineering and regenerative medicine due to their biocompatibility and controlled, sustained-release drug delivery [33]. ADSCs cultured in GS hydrogel showed better cell proliferation and differentiation [34]. A light microscopy image (Fig. 2A) showed that cells were uniformly distributed within the hydrogel, which benefit*s* biomedical applications [34]. Moreover, SEM images (Fig. 2B) confirmed that the grid-like constructs of 3D-ADSC/NO and ADSCs grew both inside the hydrogel and on the surface of the hydrogel. These results suggested that ADSCs can transplant and grow in the GS hydrogel.

Interestingly, the cumulative NO release from the hydrogel indicated a promising result involving prolonged and sustained release of NO (Fig. 2C). Previous studies have shown that the short half-life of SNAP is one of the main limitations in wound applications [35] However, our results showed that NO release exhibited a burst release within 4 h and became slow and sustained even after 120 h (up to 5 days). S-Nitrosothiols (RSNOs) are the sulphur analogues of the better-known alkyl nitrites. However, most RSNO are reported to be too unstable to isolate as pure solid and SNAP has been prepared as stable solids and has been characterized [36]. It has been known that SNAP in liquid decompose to yield NO. Therefore, incorporation of NO donors such as SNAP into various hydrogel scaffolds could be one of the best strategies to control the burst release and maintain slow and sustained release of NO [14]. The burst release of NO may be associated with the SNAP that adhered to the surface of the hydrogel, while the sustained release may be due to the swelling and degradation properties of the hydrogel. In our study, the temporal production of NO fit with the production of NO in healing wounds, which increased during the early inflammation phase and decreased during the proliferation and maturation phases [13]. The burst release of NO plays a central role in the inhibition of early inflammation [37]; slow and sustained release of NO becomes relevant during endothelial differentiation [38]. Therefore, these results indicated that the NO release of 3D-ADSCs/NO is suitable for the wound healing process.

### SNAP in the GS hydrogel had no significant inhibitory or toxic effects on ADSCs

Studies [39] have shown that different concentrations of NO produce opposite effects on stem cell behaviour. We therefore examined the cell viability and proliferation rate of ADSCs in 3D-ADSCs/NO. The Alamar Blue assay showed that the cell proliferation rate increased over time but slowly decreased after 5 days (Fig. 2D) in both 3D-ADSCs and 3D-ADSCs/NO. There was also no significant difference between 3D-ADSCs and 3D-ADSCs/NO at any time point. Furthermore, the live-dead results (Fig. 2E and F) showed that a large number of live ADSCs grew in 3D-ADSCs/NO and 3D-ADSCs even after 5 days, and there was no significant difference in cell viability between 3D-ADSCs/NO (76.9% ± 11.0%) and 3D-ADSCs (86.7% ± 18.0%), which means that SNAP had no significant toxic effects on ADSCs. The obtained results showed that SNAP had no significant inhibitory effect on the proliferation of ADSCs in 3D constructs, which was in line with previous results indicating that the 5‰ SNAP hydrogel may be the most suitable for cell viability and cell proliferation of fibroblast cells and keratinocyte cells [14]. Therefore, this 3D-ADSC/NO bioscaffold was chosen to evaluate its biological functions *in vivo* and *in vitro*.

### The 3D-ADSC/NO constructs promoted the migration and angiogenesis of HUVECs

**Figure 3.**
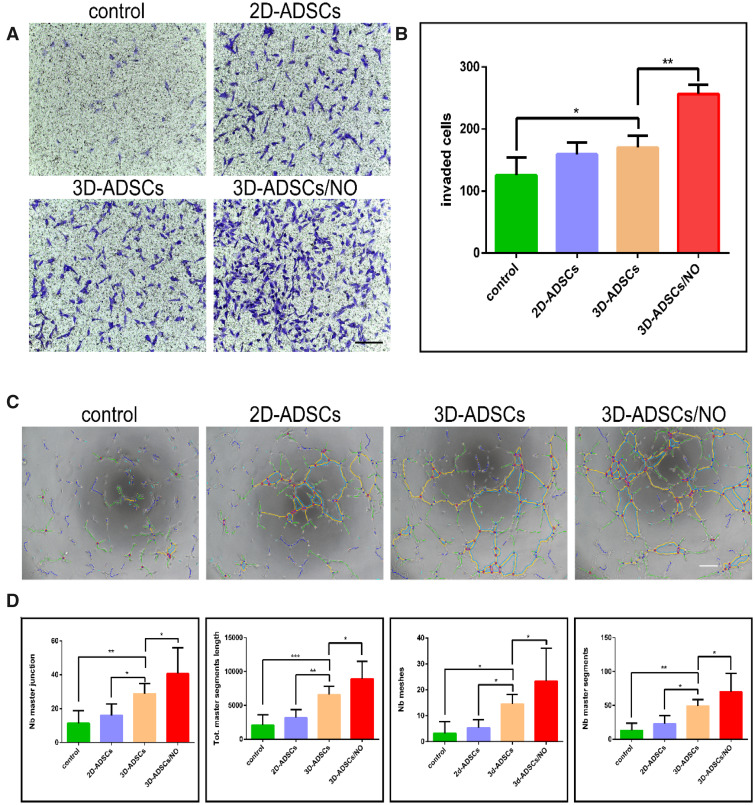
Representative pictures (**A**) and quantitative estimation (**B**) of the *in vitro* transwell assay. HUVECs stimulated by supernatant from 3D-ADSCs/NO showed a stronger migration capacity than 3D-ADSCs, 2D-ADSCs and the control group. Representative pictures (**C**) and quantitative estimation (**D**) of tubule formation assay. HUVECs stimulated by supernatant from 3D-ADSCs/NO showed a better tube-formation capacity than 3D-ADSCs, 2D-ADSCs and the control group (scale bar: 400 µm;*P < 0.05; **P < 0.01).

Traditional skin wound healing includes four classic phases: haemostasis, inflammation, proliferation and maturation. A meta-analysis showed that stem cell therapy plays a biological role in wound healing, mainly through angiogenesis and anti-inflammatory actions [40]. HUVECs have offered an important *in vitro* model for the study of angiogenesis. Therefore, HUVECs were chosen to detect the influence of 3D-ADSCs/NO on migration and angiogenesis *in vitro*. As we expected, HUVECs stimulated by supernatant from 3D-ADSCs/NO showed a stronger migration capacity than 3D-ADSCs, 2D-ADSCs and the control group, as indicated by the qualitative and quantitative results of tubule formation (Fig. 3A and B). Our results are in accordance with the report that mesenchymal stem cells from mice could promote the vascularization of HUVEC [41].

Under stimulation with 3D-ADSC/NO supernatant, the tube-formation capacity of HUVECs was better than that of the other groups (Fig. 3C and D). A previous study found that human placenta-derived mesenchymal stem cells stimulated by NO augment the angiogenic effects of HUVECs [16]. Our results also found that 3D-ADSCs/NO can enhance the migration and angiogenesis ability of HUVECs. These results suggest that the underlying mechanism of 3D-ADSCs/NO may be associated with angiogenesis.

### The 3D-ADSC/NO constructs promoted the severe burn wound healing process

**Figure 4.**
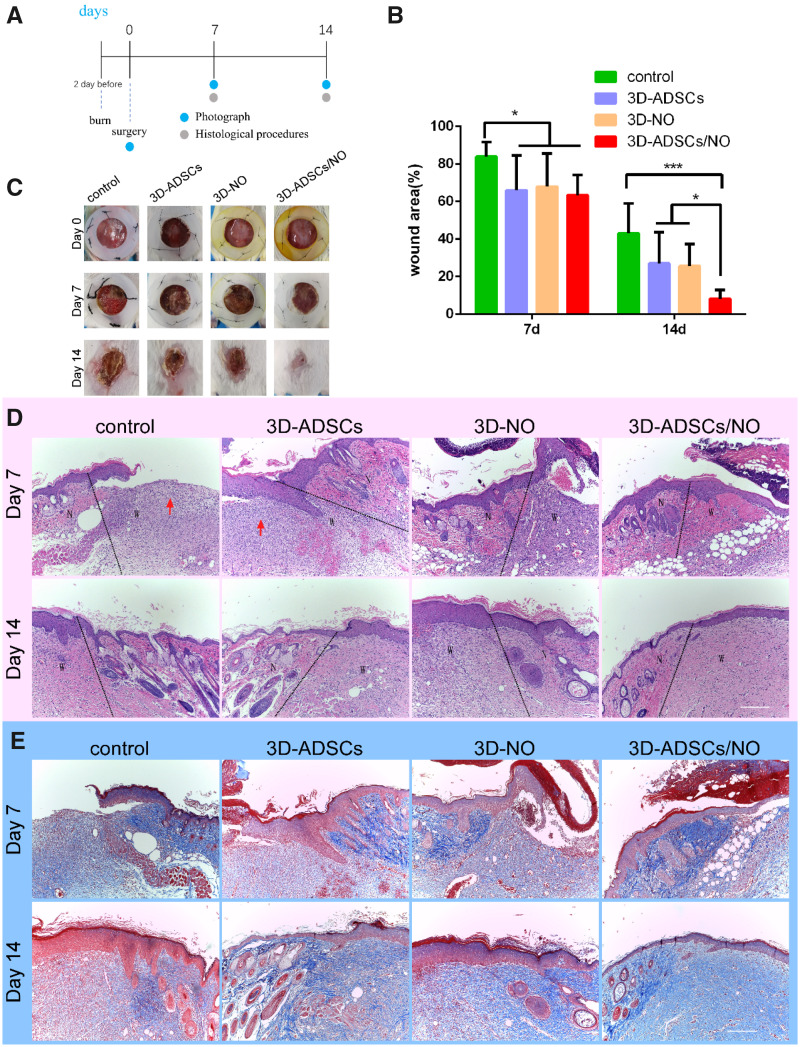
(**A**) Burn wounds were made 2 days before and the necrotic skin was removed at Day 0. Mice were randomized into four groups (*n* = 8 each group) according to different treatments after surgery: control group, 3D-ADSCs, 3D-NO and 3D-ADSCs/NO. Photograph of the wound area was performed at Days 0, 7 and 14, and mice were sacrificed to have IHC procedure at Days 7 and 14. (**B**) Quantification of wound area expressed as the percentage of the initial wound size. Wound closure can be observed at Day 14 which the 3D-ADSCs/NO group presented the most remarkable effect of wound healing. (**C**) Representative images of the mouse burn wound healing process with different treatments over 14 days. (**D**) HE-stained images of mouse burn wound skin with different treatments. Wounds treated with 3D-ADSCs/NO presented more complete skin structures. The red arrows indicate the severe inflammatory cell infiltration into the wound bed. ‘N’ is represented for normal skin and ‘w’ is represented for wound area. (**E**) MT-stained images of mouse burn wound skin with different treatments. At Days 7 and 14, the expression of collagen in wound tissue was increased in the 3D-ADSCs/NO group compared with others (*P < 0.05 and ***P < 0.001; scale bar: 200 μm).

To investigate the wound healing efficacy of 3D-ADSCs/NO *in vivo*, we generated a full-thickness skin burn model in BALB/c mice. Over time, the wound area gradually decreased, and after implantation for 14 days, 3D-ADSCs/NO (8.1% ± 4.7%) had better healing effects than 3D-ADSCs (29.3% ± 17.1%), 3D-NO (23.4% ± 10.0%) and the control group (43.1% ± 15.9%; Fig. 4B and C). 3D-ADSCs/NO showed a better tendency toward wound healing than other groups. No infection or rejection was detected during the experimental period, indicating the biocompatibility of the materials. Both alginate and gelatine have been used in a number of biomedical applications such as wound dressings, tissue engineering and drug delivery [42]. It has been reported that chitosan-poly(vinyl alcohol) hydrogels loaded with SNAP can promote angiogenesis [14]. In addition, mesenchymal stem cells cultured in 3D hydrogels accelerated wound healing through enhanced vascularization and paracrine effects in wounds [24]. The higher wound healing efficiency of the 3D cell matrix may be caused by the higher survival rate of stem cells and the hypoxic environment in the 3D constructs [43]. Mesenchymal stem cells stimulated by NO can also promote angiogenesis by enhancing the levels of VEGF and miR-126 in exosomes [16]. Furthermore, our previous study found that ADSCs can release cytokines rather than differentiate into skin cells to enhance the wound healing process [11]. A variety of growth factors (Platelet Derived Growth Factor, Fibroblast Growth Factor, Epidermal Growth Factor, VEGF etc.) and cytokines (chemokines, proinflammatory cytokines, Interleukin-10 etc.) invigorate the wound healing process [44]. Many experimental and clinical studies have demonstrated varied, but in most cases beneficial, effects of exogenous growth factors on the healing process. Thus, we suppose that 3D-ADSCs/NO can promote a fast and successful wound healing process by releasing more angiogenesis-related cytokines rather than differentiating into keratinocytes or endotheliocytes.

### Histological analysis of wound healing

As shown in Fig. 4D, at Day 7, the wounds of the control group were still ruptured, while the wounds of 3D constructs, especially 3D-ADSCs/NO, showed a more regular structure to a certain extent. The re-epithelialization was accelerated by treatment with 3D-ADSCs/NO. Moreover, there was severe inflammatory cell infiltration into the wound bed of the control group and 3D-ADSCs at Day 7 (red arrow), which was not shown in 3D-ADSCs/NO and 3D-NO. This could be due to the anti-inflammatory action of NO, which can regulate cytokines that initiate inflammation (e.g. interleukins, monocytes and neutrophils) [45]. Excessive/prolonged inflammation may inhibit wound healing and an anti-inflammatory dressing can accelerate wound healing [46]. In addition, re-epithelialization is an essential event during the wound healing process. All groups showed complete epithelialization at 14 days; however, 3D-ADSCs/NO presented more complete skin structures, whereas other groups showed irregular epidermal hyperplasia and hyperkeratosis. Specifically, treatment with 3D-ADSCs/NO resulted in more organized connective tissue than that of the other groups.

In addition to re-epithelialization, collagen deposition is another necessary event during the wound healing process. As shown in Fig. 4E, the collagen deposition on the wound bed of 3D-ADSCs/NO was relatively denser and more continuous than that of the other three groups. The semiquantitative analysis (Supplementary Fig. S1B) of collagen deposition found that 3D-ADSCs/NO showed a significantly higher amount of collagen than the control group, 3D-ADSCs and 3D-NO at both 7 and 14 days. The histological results from our study are in agreement with previous research showing that ADSCs can enhance collagen deposition in burn wounds [9]. The interaction between NO and ADSCs resulted in better wound-healing effects than the 3D-ADSC and 3D-NO groups.

### The 3D-ADSC/NO constructs enhanced angiogenesis *in vivo*

**Figure 5.**
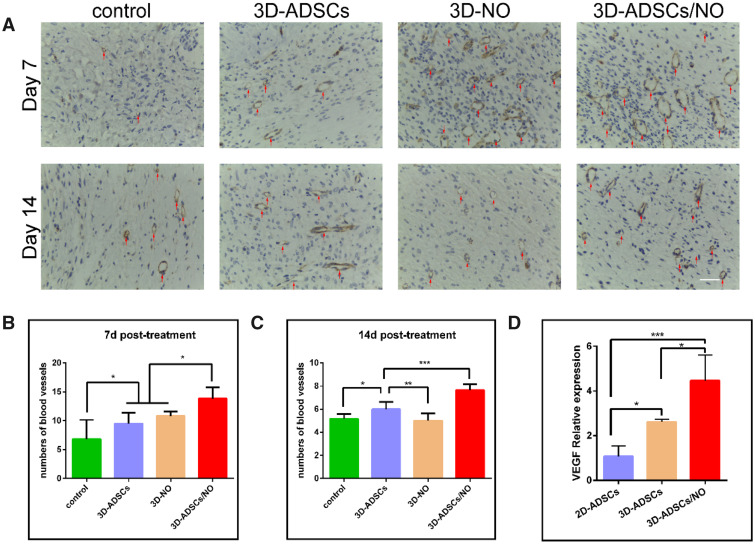
(**A**) CD31 Staining of mouse burn wound skin with different treatments at 7 and 14 days. More mature blood vessels were observed in the 3D-ADSCs/NO group than in the control, 3D-ADSCs and 3D-NO groups. Quantitative comparison of CD31-positive (+) blood vessels at 7 days (**B**) and 14 days (**C**) between different treatment groups (scale bar: 40 µm). (**D**) Statistical analysis for the expression of VEGF (*P < 0.05; **P < 0.01; and ***P < 0.001).

Angiogenesis plays an important role in wound repair, and the condition of new vessel formation reflects healing status [47]. We performed immunohistochemical staining of CD31 to detect angiogenesis in skin tissues (Fig. 5A). Quantitative analysis (Fig. 5B and C) showed that, at both 7 and 14 days, more mature blood vessels were observed in the 3D-ADSCs/NO group than in the control, 3D-ADSCs and 3D-NO groups (*P* < 0.001). Both NO therapy [19] and stem cell therapy [48] can promote wound healing by stimulating endothelial cells. The increased number of blood vessels in 3D-ADSCs/NO may be due to the angiogenic effect of more cytokines secreted by ADSCs under hydrogel and NO addition, which can enhance the neogenesis and maturation of capillaries.

### The angiogenesis ability of ADSCs in 3D-ADSCs/NO may be associated with the secretion of VEGF

With the discovery of VEGF more than 30 years ago and its therapeutic potential for vascular therapy, it was considered the most important growth factor in angiogenesis [49]. Meanwhile, some studies indicate that both NO and ADSCs may initiate regrowth of epithelial cells and close open wounds through the VEGF signalling pathway [16]. To investigate the potential signalling pathway of 3D-ADSCs/NO in burn wound healing, the expression of VEGF in ADSCs was analysed. As shown in Fig. 5D, the critical proangiogenic factor VEGF was obviously upregulated in 3D-ADSCs/NO, which is consistent with the above results and previous research by others [14, 16]. Study has also shown that hydrogel-based biomaterials have porous structures that allow for the diffusion of signalling molecules [50]. NO has been reported to play an important role in cGMP-PKG signalling pathway [51]. Recent studies indicated that cGMP-PKG signalling pathway can promote the autophagy [52]. Autophagy is a cellular protective process and maintains cellular homeostasis under starvation or hypoxia conditions. Furthermore, autophagy has been shown to enhance MSC-mediated vascularization via regulation of VEGF secretion [53]. We suspect that NO can enhance the autophagy of ADSCs through Cyclic Guanosine Monophosphate-Protein Kinase G (cGMP-PKG) signalling pathway in 3D constructs and the secretion of VEGF in ADSCs. However, further research should clarify this point. Our research first indicated that 3D-printing ADSCs and NO bioscaffolds maximized the therapeutic potential of stem cell and promoted the wound healing via the VEGF signalling pathway. According to our research results, the combination of ADCSs and NO can promote burn wound healing. Therefore, the future trends in clinical translation may lie as following: after debridement of massive injury and isolated autologous ADSCs, the ADSCs and NO can be applied to the burn wound using *in situ* 3D printing technology. The cytokines derived from ADSCs and NO could promote the wound healing with a better vascularization.

## Conclusion

The integral 3D-ADSC/NO hydrogel scaffolds were beneficial to cell growth and promoted the angiogenesis of HUVECs *in vitro.* 3D-ADSCs/NO exhibited effective wound healing by stimulating collagen expression and angiogenesis in a severe burn model and may involve the VEGF signalling pathway. Herein, 3D-ADSC/NO constructs may be a novel therapeutic approach for severe burn wound healing.

## Supplementary Material

rbab014_Supplementary_Data
