# Supplementary material for: 3D bioprinting of integral ADSCs-NO hydrogel scaffolds to promote severe burn wound healing
Source: Regen Biomater. 2021 Apr 25;8(3):rbab014. doi: 10.1093/rb/rbab014 (PMC8071097; doi:10.1093/rb/rbab014)
Supplement: rbab014_Supplementary_Data [file rbab014_supplementary_data.docx]

**Dear *Regenerative Biomaterials* Editors:**

Thank you for giving us the opportunity to submit a revised draft of the manuscript titled “*3D bioprinting of integral ADSCs-NO hydrogel scaffolds to promote severe burn wound healing*.” (ID: RB-2020-147).

We appreciate the time and effort that you and the reviewers have dedicated to providing your constructive comments on our manuscript. The received comments are valuable and constructive to our research. Accordingly, we have studied the suggestions carefully and have revised it according to the journal guidelines. Please find that all the changes were highlighted in red in the manuscript. Please find point-by-point responses are listed below this letter. We hope that the revised manuscript is now acceptable for publication in your journal.

Looking forward to hearing from you soon.

With kindest regards,

Yours Sincerely

**Lei Zhu**, Corresponding author

E-mail: zhulei@mail.sysu.edu.cn

**Tao Xu,** Corresponding author

E-mail: taoxu@mail.tsinghua.edu.cn

***Point-by-point Response to Reviewers***

*First of all, we thank both reviewers and editor for their positive and constructive comments and suggestions.*

Replies to Reviewer #1:

1. -Abstract
   Information about what in vivo model applied in the study is needed.

Response: Thank you for pointing this out. We provide the information about in vivo model in Abstract.

We added this point into our revised manuscript and the details can be found in **Line 21-25, Page 2**: “*Burn wound healing experiments in mice revealed that 3D-ADSCs/NO accelerated the wound healing by promoting faster epithelialization and collagen deposition*.”

2. -Introduction
   Whether there are clinical studies/trials related to ADSCs or NO in burn injury?

Response: Thank you for this comment. There is no doubt that stem-cell research in the field of Burns Surgery has yet to yield any groundbreaking results. However, ADSCs-based therapy has achieved very good surgical outcomes and patient satisfaction in burn wound^[1]^. Furthermore, a multicenter, randomized controlled, observer-blinded study has shown that continuous NO supplement improve healing in foot ulcers of patients with diabetes^[2]^. At present, few studies explore the interaction between ADSCs and NO in burn injury.

We added this point into our revised manuscript and the details can be found as follows:

**Line 27-29, Page 3**: “*In addition, ADSCs have been permitted to be used in clinical and achieved very good surgical outcomes and patient satisfaction in burn wound [10]*.”

**Line 41-43, Page 3**: “*In many chronic ulcers, and especially in patients with diabetes, the continuous supplement of NO improve healing process. [15]*”

3. -Methods
   The differentiation ability of ADSCs should be provided.

Response: Thank you for this constructive comment. ADSCs were isolated following standard protocol^[3]^. And we had characterized the differentiation ability of ADSCs according to our previous study^[4]^. Differentiation abilities of the isolated cell were examined by adipogenic, osteogenesis, and chondrogenic. Staining of Oil Red O, Alizarin Red, and Alcian blue was carried out to verify the differentiation capacity. Flow cytometry analysis also identified the ADSCs.

We added this point into our revised manuscript and the details can be found in **Line 56-58, Page 4：“***We had identified the ADSCs through flow cytometry analysis and differentiation abilities analysis in our previous study[27].*”

What are the culture methods for the HUVECs in the study?

Response: Thank you for this comment. HUVECs were cultured following the standard protocol. Specifically, Human Umbilical Vein Endothelial Cells (HUVECs) were cultured in DMEM supplemented with 10% FBS in a humidified atmosphere containing 5% CO2 at 37°C.

We added this point into our revised manuscript and the details can be found in **Line 33-35, Page 6:**“*HUVECs were cultured in DMEM supplemented with 10% FBS in a humidified atmosphere containing 5% CO2 at 37°C.*”

Why choose HUVECs to study the interaction within the bioscaffold? Is there any immunosuppression between ADSCs from mice and HUVECs from the human species?

Response: Thank you for this comment. Angiogenesis involves the proliferation, migration, and remodeling of endothelial cells in the process of tube formation. HUVECs have offered an important in vitro model for the study of all three of these processes^[5]^. Culture of HUVECs on Matrigel results in the formation of honeycomb-like structures that simulate tube formation by endothelial cells in vivo^[6]^ and culture of HUVECs on Transwell simulate the cell migration in vivo^[7]^.

Mesenchymal stem cells are the most commonly used cell-based therapy in clinical trials because of their regenerative effects, ease of isolation, and low immunogenicity^[8]^. Previous study also shown that mesenchymal stem cells from mice could promote the vascularization of HUVEC^[9]^. We use the culture supernatant of ADSCs to investigate the interaction of ADSCs and HUVEC, so the low immunosuppression between ADSCs and HUVEC do not appear to affect our conclusions.

We added this point into our revised manuscript and the details can be found as follows:

**Line 29-31, Page 10:** “*HUVECs have offered an important in vitro model for the study of angiogenesis.*”

**Line 39-41, Page 10:**“*Our results are in accordance with the report that mesenchymal stem cells from mice could promote the vascularization of HUVEC[41].*”

Please provide a detailed in vivo experimental flow chart. What is the effect of alginate hydrogel alone for the burn models?

Response: Thank you for this constructive comment. We added a detailed in vivo experimental flow chart in Figure 4A.

Both alginate and gelatin have been used in a number of biomedical applications such as wound dressings, tissue engineering and drug delivery^[10]^. Previous study also show that Alginate/Gelatin hydrogel improves wound healing^[11]^. The use of alginates as dressings stems primarily from their ability to absorb the wound secretions.^[12]^ Further experiments would be required to rule out the influence of alginate hydrogel in burn wound. However, the result of burn wound still proved the combination of ADSCs and NO was better than ADSCs alone or NO alone.

4. -Results and discussion
   The results and discussion section should be expanded to provide readers the uniqueness of the authors' findings and how this research can be further explored to achieve clinical utility.
   The manuscript would be strengthened if the authors further discuss the possible molecular interaction between ADSCs and NO.

Response: Thank you for the reviewer’s constructive suggestion. We rewrote several paragraphs and added in some discussions involved in-depth mechanism.

Details can be found in **Line 19-47, Page 13**: “*NO has been reported to play an important role in cGMP-PKG signaling pathway [51]. Recent studies indicated that cGMP-PKG signaling pathway can promote the autophagy [52]. Autophagy is a cellular protective process and maintain cellular homeostasis under starvation or hypoxia conditions. Furthermore, autophagy has been shown to enhance MSC-mediated vascularization via regulation of VEGF secretion [53]. We suspect that NO can enhance the autophagy of ADSCs through cGMP-PKG signaling pathway in 3D constructs and the secretion of VEGF in ADSCs. However, further research should clarify this point.* *Our research first indicated that 3D-printing ADSCs and NO bioscaffolds maximized the therapeutic potential of stem cell and promoted the wound healing via the VEGF signaling pathway. According to our research results, the combination of ADCSs and NO can promote burn wound healing. Therefore, the future trends in* *clinical translation may lie as following: after debridement of massive injury and isolated autologous ADSCs, the ADSCs and NO can be applied to the burn wound using in situ 3D printing technology. The cytokines derived from ADSCs and NO could promote the wound healing with a better vascularization.”*

5. -Others:
   The term "immunofluorescence" should be corrected to "immunohistochemistry" on line 25 of page 2.
   "Nitric oxide (NO)" should be abbreviated to "NO" on line 39 of page 3.
   Figure legends should be more descriptive and point out the importance of the data.
   Fig3. Please highlight the tube end of the tubule formation.
   Fig4. Please highlight the indicating results.  
   Fig3, 5. Scale bar is indistinct.

Response: Thank you for the careful review. We agree with those suggestions. We fixed the spell errors

Details can be found as follows:

**Line 25, Page 2**: “*Notably, immunohistochemistry of CD31 suggested an increase in neovascularization, supported by the upregulation of vascular endothelial growth factor (VEGF) mRNA in ADSCs in the 3D biosystem*”.

**Line 39, Page 3**: “*NO has become an attractive candidate…*”

And we rewrote the figure legends and made some revisions to the figures. We highlight the results of tubule formation in Fig.3 and the indicating results in Fig.4. The scale bar in Fig3,5 has been modified.

Replies to Reviewer #2:

1. The hydrogel with SNAP shows controlled and sustained release of NO. The way that SNAP release NO and how the gelatin-alginate hydrogel can change the burst release of NO should be well explained in the text.

Response: Thank you for the careful review. S-Nitrosothiols (RSNO) are the sulfur analogues of the better-known alkyl nitrites. However, most RSNO are reported to be too unstable to isolate as pure solid and SNAP has been prepared as stable solids and has been characterized^[13]^. It has been known that RSNO decompose to yield the corresponding disulfide and NO: $RSNO\to\frac{1}{2}RSSR+NO$. SNAP becomes unstable in water. Therefore, incorporation of NO donors such as SNAP into various hydrogel scaffolds could be one of the best strategies to control the burst release and maintain slow and sustained release of NO^[14]^. The burst release of NO may be associated with the SNAP that adhered to the surface of the hydrogel. And the sustained release of NO may due to the swelling and degradation of the hydrogel^[14]^.

We added this point into our revised manuscript and the details can be found in **Line 17-29, Page 9**: “*S-Nitrosothiols (RSNO) are the sulfur analogues of the better-known alkyl nitrites. However, most RSNO are reported to be too unstable to isolate as pure solid and SNAP has been prepared as stable solids and has been characterized [36]. It has been known that SNAP in liquid decompose to yield NO. Therefore, incorporation of NO donors such as SNAP into various hydrogel scaffolds could be one of the best strategies to control the burst release and maintain slow and sustained release of NO [14].*”

2. Some details about 3D bio-printing should be described.
   (1) This work prepares a viscous solution containing 20% gelatin and 2% alginate for bioprinting. Air bubbles can easily get in the solution during the process of mixing the cell supernatant with hydrogel solution. How can air bubbles be remove without harming cells?

Response: Thank you for this comment. In our research, the cell supernatant was mixed with hydrogel solution through gentle blowing and beating to avoid air bubbles. Besides, air bubbles can be removed with 37℃ aqueous bath 2min. Our 3D scaffolds had a small volume (about 0.3ml each scaffold), so there were not many air bubbles. In the further clinical application of this research, vacuum degassing may be considered.

(2) The parameters for 3D bio-printing should be written in the experiment part, including temperature, speed, pressure, needle diameters, layers, printing pathway.

Response: In our research, the square grid scaffolds were designed with 15×15mm cross sectional area and 2mm thickness. The printing chamber was set to 4℃, pressure was set to 0.2mPa, 0.26 mm diameter nozzle was selected, and the scanning speed was controlled at 8mm/s. The printing pathway was grid-like and each layer had 8 microwires.

We added this point into our revised manuscript and the details can be found in **Line 17-26, Page 5**: “*The square grid scaffolds were designed with 15×15mm cross sectional area and 2mm thickness. The printing chamber was set to 4℃, pressure was set to 0.2mPa, 0.26 mm diameter nozzle was selected, and the scanning speed was controlled at 8mm/s. The printing pathway was grid-like and each layer had 8 microwires.*”

1. Arkoulis N, Watson S, Weiler-Mithoff E. **Stem cell enriched dermal substitutes for the treatment of late burn contractures in patients with major burns**. *Burns* 2018; 44(3):724-726.

2. Edmonds ME, Bodansky HJ, Boulton AJM, Chadwick PJ, Dang CN, D'Costa R, et al. **Multicenter, randomized controlled, observer-blinded study of a nitric oxide generating treatment in foot ulcers of patients with diabetes-ProNOx1 study**. *Wound repair and regeneration : official publication of the Wound Healing Society [and] the European Tissue Repair Society* 2018; 26(2):228-237.

3. Zhang J, Liu Y, Chen Y, Yuan L, Liu H, Wang J, et al. **Adipose-Derived Stem Cells: Current Applications and Future Directions in the Regeneration of Multiple Tissues**. *Stem cells international* 2020; 2020:8810813.

4. Luo Y, Yi X, Liang T, Jiang S, He R, Hu Y, et al. **Autograft microskin combined with adipose-derived stem cell enhances wound healing in a full-thickness skin defect mouse model**. *Stem Cell Research & Therapy* 2019; 10(1).

5. Park HJ, Zhang Y, Georgescu SP, Johnson KL, Kong D, Galper JB. **Human umbilical vein endothelial cells and human dermal microvascular endothelial cells offer new insights into the relationship between lipid metabolism and angiogenesis**. *Stem cell reviews* 2006; 2(2):93-102.

6. Li X, Wang Y, Shi L, Li B, Li J, Wei Z, et al. **Magnetic targeting enhances the cutaneous wound healing effects of human mesenchymal stem cell-derived iron oxide exosomes**. *Journal of nanobiotechnology* 2020; 18(1):113.

7. Sun J, Shen H, Shao L, Teng X, Chen Y, Liu X, et al. **HIF-1alpha overexpression in mesenchymal stem cell-derived exosomes mediates cardioprotection in myocardial infarction by enhanced angiogenesis**. *Stem Cell Res Ther* 2020; 11(1):373.

8. Moreira A, Kahlenberg S, Hornsby P. **Therapeutic potential of mesenchymal stem cells for diabetes**. *Journal of molecular endocrinology* 2017; 59(3):R109-r120.

9. Chen Z, Wang H, Xia Y, Yan F, Lu Y. **Therapeutic Potential of Mesenchymal Cell-Derived miRNA-150-5p-Expressing Exosomes in Rheumatoid Arthritis Mediated by the Modulation of MMP14 and VEGF**. *Journal of immunology (Baltimore, Md : 1950)* 2018; 201(8):2472-2482.

10. Balakrishnan B, Mohanty M, Umashankar PR, Jayakrishnan A. **Evaluation of an in situ forming hydrogel wound dressing based on oxidized alginate and gelatin**. *Biomaterials* 2005; 26(32):6335-6342.

11. Diniz FR, Maia R, Rannier L, Andrade LN, M VC, da Silva CF, et al. **Silver Nanoparticles-Composing Alginate/Gelatine Hydrogel Improves Wound Healing In Vivo**. *Nanomaterials (Basel, Switzerland)* 2020; 10(2).

12. Boateng JS, Matthews KH, Stevens HN, Eccleston GM. **Wound healing dressings and drug delivery systems: a review**. *Journal of pharmaceutical sciences* 2008; 97(8):2892-2923.

13. Butler AR, Rhodes P. **Chemistry, analysis, and biological roles of S-nitrosothiols**. *Analytical biochemistry* 1997; 249(1):1-9.

14. Zahid AA, Ahmed R, Raza Ur Rehman S, Augustine R, Tariq M, Hasan A. **Nitric oxide releasing chitosan-poly (vinyl alcohol) hydrogel promotes angiogenesis in chick embryo model**. *Int J Biol Macromol* 2019; 136:901-910.
